# Implementing a Digital Mental Health Intervention—the Lumi Nova App—to Support Children With Anxiety in Economically Disadvantaged Areas: Mixed Methods Study

**DOI:** 10.2196/60611

**Published:** 2025-10-14

**Authors:** Pauline Whelan, Heidi Tranter, Lesley-Anne Carter, John Sainsbury, Manjul Rathee, Charlotte Stockton-Powdrell, Niamh Bolton, Kathryn Mary Abel

**Affiliations:** 1 Division of Informatics, Imaging and Data Science, School of Health Sciences, Faculty of Biology, Medicine and Health, University of Manchester, The Christabel Pankhurst Institute, Oxford Road, Manchester, M139PT, United Kingdom, 44 161 306 6000; 2 GM.Digital Research Unit, Greater Manchester Mental Health NHS Foundation Trust, Manchester, United Kingdom; 3 Centre for Women’s Mental Health, Faculty of Biology, Medicine and Health, University of Manchester, Manchester, United Kingdom; 4 Centre for Biostatistics, Division of Population Health, Health Services Research and Primary Care, Faculty of Biology, Medicine and Health, University of Manchester, Manchester, United Kingdom; 5 Research and Innovation, Greater Manchester Mental Health NHS Foundation Trust, Manchester, United Kingdom; 6 BFB Labs, London, United Kingdom

**Keywords:** digital mental health, anxiety, digital intervention, children, mHealth, socio-economic disadvantage, deprivation, child, adolescent, youth, Lumi Nova, app, economically-disadvantaged areas, mixed method study, quantitative, qualitative, semi-structured interview, web-based, mobile health, health informatics, digital mental health intervention

## Abstract

**Background:**

Anxiety is one of the most common mental health problems experienced by children worldwide. In the United Kingdom, many children experiencing anxiety do not receive adequate or timely help. Children living in economically disadvantaged areas experience more mental health problems than those living in high-income areas and are less able to engage in activities that can have a positive or protective impact on their mental health. The need for providing low-cost, accessible, and engaging mental health interventions for children living in these areas is high.

**Objective:**

The study aimed to explore how a digital mental health therapeutic, “Lumi Nova: Tales of Courage” (BfB Labs Ltd), could be used to support children living with anxiety in economically disadvantaged areas.

**Methods:**

A mixed method study design was used to explore the implementation of Lumi Nova using a supported delivery model with mental health teams based in the North of England. Quantitative data collection on recruitment and engagement patterns were collected and analyzed. Qualitative research explored children’s, parents’, and practitioners’ views and experiences with the Lumi Nova app.

**Results:**

113 children consented to use Lumi Nova and 98 (87%) accessed the intervention at least once. Qualitative semistructured interviews found that children, their parents, and practitioners viewed the Lumi Nova app positively. Quantitative analysis of the recruitment data suggested the feasibility of a future larger roll-out. Analysis of usage data demonstrated varied patterns of engagement with the intervention. The frequency and duration of usage varied across children, as did the activities completed within the game: almost half (49%) completed 3 in-game challenges, indicating progression through the treatment pathway.

**Conclusions:**

The study demonstrated that a digital mental health intervention could be successfully deployed within economically disadvantaged areas in the United Kingdom to support children experiencing anxiety. Expected barriers to the deployment of digital mental health interventions in economically disadvantaged areas (eg, lack of access to smartphones, data plans, and lack of technical skills) were not reported. Digital mental health interventions have the potential to address current gaps in mental health provision for disadvantaged individuals and communities.

## Introduction

Approximately 1 in 5 children and young people aged 8‐25 years in the United Kingdom had a probable mental disorder in 2023; more than 20% of these children and young people were between the ages of 8 and 16 years [1]. Anxiety is one of the most common psychological symptoms and one of the most common mental illnesses worldwide, and it often starts by the age of 13 years [2]. Anxiety and depression are also the most common mental disorders experienced by children in the United Kingdom [3]. Despite the existence of effective anxiety treatments in England, many children and young people do not receive appropriate, evidence-based, or timely support. Greater demands on child and adolescent mental health services (CAMHS), combined with parents’ difficulties about where and how to access appropriate support, are key barriers for families trying to access help [4].

A recent study explored the effectiveness of delivering low-intensity cognitive behavioral interventions to children and young people between the ages of 6 and 18 years for support with sleep [5]. Results showed low-intensity interventions delivered by trainee practitioners could improve mental health, anxiety, and depression symptom severity and reduce the number of children and young people who reach the clinical threshold for mental health disorders [5]. However, one of the limitations of this study is both the cost (both financial and resource) and the feasibility of trainee practitioners providing this early help intervention across the United Kingdom. There is a need for low-cost, sustainable solutions that do not rely significantly on staff time, which is already stretched.

Such barriers are especially problematic for children and young people from economically disadvantaged backgrounds, who are likely to be disproportionately affected by the challenges of accessing timely support. In the United Kingdom, children living in households in the lowest 20% income bracket are 4 times more likely to experience mental illness compared to those in the highest 20% income bracket [6]. Overall, 26.8% of children aged 8‐16 years with a probable mental disorder were unable to take part in activities outside of school or college as their parents were unable to afford these [1]. Engaging in such activities has been well documented to have a positive influence on the mental health, well-being [7], and resilience of young people [8]. The combination of deprivation, low parental education [9], and length of time taken to seek and access support [10] means that more timely, accessible interventions are urgently needed for children and young people, and especially those from disadvantaged families.

As “demand” for mental health services outstrips “supply,” digital technologies have been proposed to replace or supplement traditional modes of therapy. Digital tools are seen as a way to plug the gaps in traditional mental health service provision by providing accessible, low-cost treatment that can be deployed at scale [11]. Children and young people are often considered an ideal population for targeting digital mental health interventions because of their comfort and familiarity with digital technologies and dense smartphone usage. In a sample of children from across the United Kingdom, around two-thirds of 3‐ to 17-year-olds used mobile phones to access the internet and, by the age of 12 years, ownership of a smartphone was almost universal [12]. A systematic review exploring children and young people’s engagement with digital mental health interventions concluded that text-light, video-rich solutions were preferred [13]. These authors also suggested a retention rate of almost 80%, considerably higher than retention rates of children and young people reported elsewhere, between 33.3% and 69.6% [14]. Development of digital interventions tailored specifically to the needs of young people is especially important in the field of mental health [13] to improve engagement with interventions and optimize their potential benefits. There is also a need for interventions targeted at younger children, that is, not adolescents, who may be more likely to be supported by parents to engage and have improved retention rates.

Of the many digital interventions available to support children with anxiety, few have been evaluated formally or empirically [11,15,16]. Where studies have been reported, they have typically not focused on working with economically disadvantaged children and young people. This is important because they may have different needs and problems of digital exclusion (lack of access to devices, data plans, and low technical literacy) are often reported to be higher in economically disadvantaged areas [17]. The current study aimed to explore how an evidence-informed therapeutic mobile game could be implemented to support primary school children from economically disadvantaged areas. For the purposes of the study, we operationalized “economically disadvantaged” as children living in households from a postcode in the lowest 3 deciles of deprivation as rated by the Index of Multiple Deprivation [18] or were in receipt of free school meals [19].

**Figure 1.**
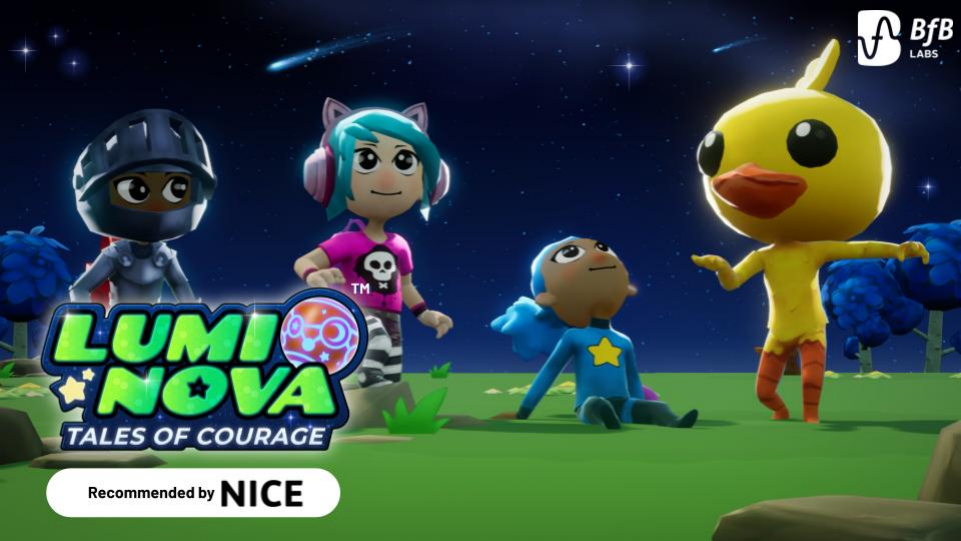
Lumi Nova gameplay.

“Lumi Nova: Tales of Courage” (BfB Labs Ltd) is a fun, engaging therapeutic digital intervention providing timely access to evidence-based support to children facing difficulties with anxiety (see Figure 1 for gameplay). It was launched in September 2020 and has been used by mental health providers in the United Kingdom to support children and young people who experience anxiety. It is a smartphone app for children aged 7‐12 years that facilitates psychoeducation and graded exposures, the main active ingredient of Cognitive Behavioral Therapy (CBT). Graded exposure is included in the National Institute for Health and Care Excellence (NICE) recommendations for supporting children to manage their worries [20]. The app is recommended by NICE (Early Value Assessment [EVA]) as a first-line treatment option for anxiety in children while further real-world evidence is generated. Lumi Nova combines ethical and studio quality immersive gaming with exposure therapy to provide instant access to therapeutic “best practice” in a way that is practical, age appropriate, nonstigmatizing, encourages self-management, and provides user progress and health outcomes data in real time to professionals. It has been developed as a result of extensive cocreation with experts from the University of Reading, MindTech (National Institute for Health and Care Research funded medtech cooperative), clinicians, teachers, parents, and children.

It is registered as a Class 1, low-risk medical device with the United Kingdom’s Medicines and Healthcare products Regulatory Agency (MHRA), has obtained a Conformité Européenne (CE) mark, and has approval from the National Health Service (NHS) for the NHS Digital Technology Assessment Criteria (DTAC). It is currently being rolled out in different parts of the United Kingdom, funded by the NHS and voluntary sector organizations. Lumi Nova is designed to enable children and young people to self-manage their fears and worries by allowing them to engage in relevant simulations for graded exposure to their fears. It consists of 15 unique goals to support children with social anxiety, separation anxiety, and phobias, with each goal containing a range of 7‐10 exposure challenges. Lumi Nova enables real-time engagement, progress updates, and outcome data for partner sites to monitor the progress of children using the intervention [21].

Lumi Nova uses a CBT-based approach while focusing on the most effective therapeutic components within it, namely psychoeducation and exposure therapy. Psychoeducation helps the child or young person to recognize and normalize their feelings of worry and understand the link between their thoughts, feelings, physical sensations, and behaviors. Exposure therapy breaks down fears and worries by exposing oneself to those feared situations, which helps children and young people learn they can cope with the fear they experience.

For the first time in this study, we implemented Lumi Nova to support children and young people from economically disadvantaged areas who were experiencing difficulties with anxiety. This study explored if Lumi Nova would be usable and acceptable to these children and young people; to understand their patterns of usage and their overall engagement with the Lumi Nova platform. We also included criteria that would be useful for demonstrating the feasibility of a future randomized control trial (eg, rates of recruitment and retention). Our primary aim was to understand the barriers and enablers to implementing a digital intervention for a population often overlooked in digital mental health studies.

## Methods

### Study Design

This was a mixed-methods study using quantitative data on recruitment and engagement to understand patterns of uptake and usage of Lumi Nova, and descriptive statistics of participant demographics. Recruitment and retention participant data was collected and analyzed to help assess the feasibility of a future larger study. Qualitative analysis was used to explore the experiences and perspectives of using Lumi Nova. Semistructured interviews were conducted with 11 professionals, 7 children, and 7 parents to explore the usability and acceptability of Lumi Nova and any barriers and enablers to the implementation of the digital technology.

### Participants

Lumi Nova is an intervention for children aged between 7 and 12 years of age who are experiencing symptoms of anxiety. Participants were eligible for inclusion if they were: from a postcode in the lowest 3 deciles of deprivation as rated by the Index of Multiple Deprivation [18] or registered for free school meals; aged 7‐12 years at the time of approach for consent; had a parent or guardian involved as a point of contact with the practitioner; and experiencing symptoms of anxiety. Initially, participants could only be included if they came from a postcode in the lowest 2 deciles; however, the limit was increased to the lowest 3 deciles because it was challenging to recruit sufficient participants within the timeframe of the study otherwise. Children were not eligible if they were deemed “in crisis” by the recruiting mental health practitioner. The exclusion criteria did not restrict children who did not have access to a smartphone from participating in the study, as devices were available for loan.

A full-time Research Digital Navigator was appointed at the lead mental health trust and managed by the Trust Innovation Lead to support the running of the study across multiple sites. The Research Digital Navigator presented Lumi Nova to multiple schools across 2 areas of socioeconomic deprivation in the Northwest of England. Schools were selected based on their location within a deprived area and based on existing links with mental health staff within the schools. Education Mental Health and Wellbeing practitioners and CAMHS staff working in child and adolescent mental health services identified children from these schools who were eligible to participate based on the above inclusion and exclusion criteria and supported referrals from primary schools into their service. The services involved in recruitment included: 2 core CAMHS teams (NHS mental health services for children and young people who assess and treat mental health difficulties); 2 mental health schools teams (generally NHS professionals who provide direct mental health support to education provisions); and “other school support” which refers to mental health professionals working with schools and educational staff to both identify and support mental health difficulties within schools, and to offer earlier interventions to families through the most appropriate services. As these services have various roles, the involvement of these services in recruiting and supporting children and young people and their families through the Lumi Nova treatment pathway was a critical element in the study’s design. The close proximity of these services to schools, contact with a high volume of referrals for anxiety, and parental involvement were integral aspects of their therapeutic modus operandi. The Research Digital Navigator completed formal consent conversations with parents and assent conversations with the participating children.

**Figure 2.**
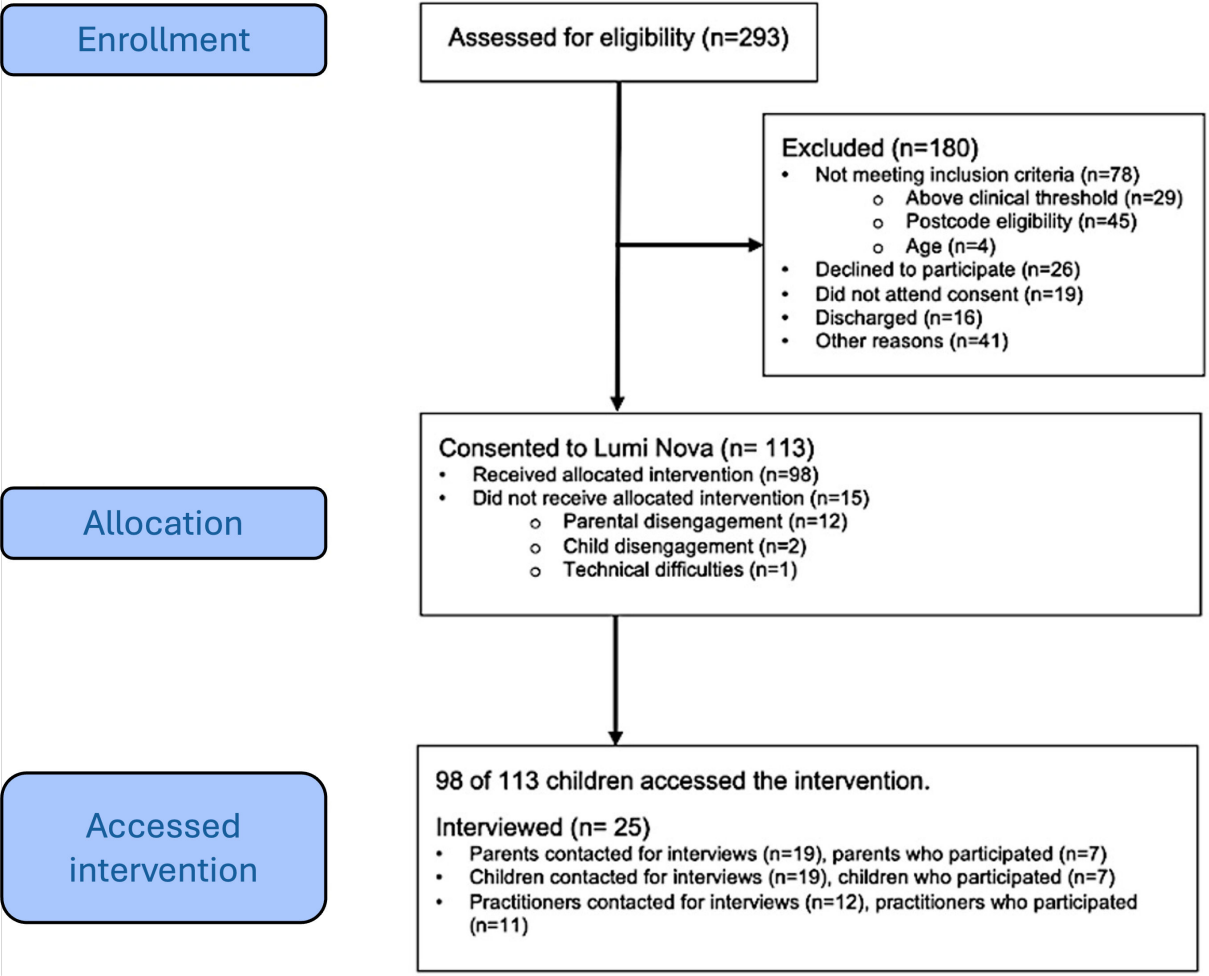
CONSORT (Consolidated Standards of Reporting Trials) flow diagram.

Overall, we recruited 113 children aged 7‐12 years from 2 regions in the North of England (Figure 2). Recruitment was led by the Innovation Lead and Research Digital Navigator based at a northern NHS Trust, who liaised with 47 Trust-employed practitioners. The local National Institute for Health and Care Research (NIHR) Clinical Research Network also promoted the study at a strategic level across local education departments and through the Healthy Schools Network. Information was shared through posters and slide decks, which were presented to schools and mental health teams, and at bespoke engagement sessions. Primary schools and mental health teams collaborated with the research team to identify appropriate children for the intervention. The school then completed a referral form for the child to access Lumi Nova, with oversight from the local mental health team and support from the Research Digital Navigator. Children and young people were identified by staff across 30 schools. Of those children, parents, and practitioners already involved in the study, 25 participated in follow-up semistructured interviews to further understand their experience of Lumi Nova (for the breakdown of interviewees, see Figures2  3).

After consenting to the study, children were given access to Lumi Nova for an 8-week period. The initial onboarding process was supported by parental or guardian engagement and a guided walkthrough with a mental health practitioner. Usage and engagement analytics, as well as in-game scores (measuring anxiety), were collected automatically through the mobile app and pushed to a secure online portal, the “VitaMind Hub,” accessed by the research team.

**Figure 3.**
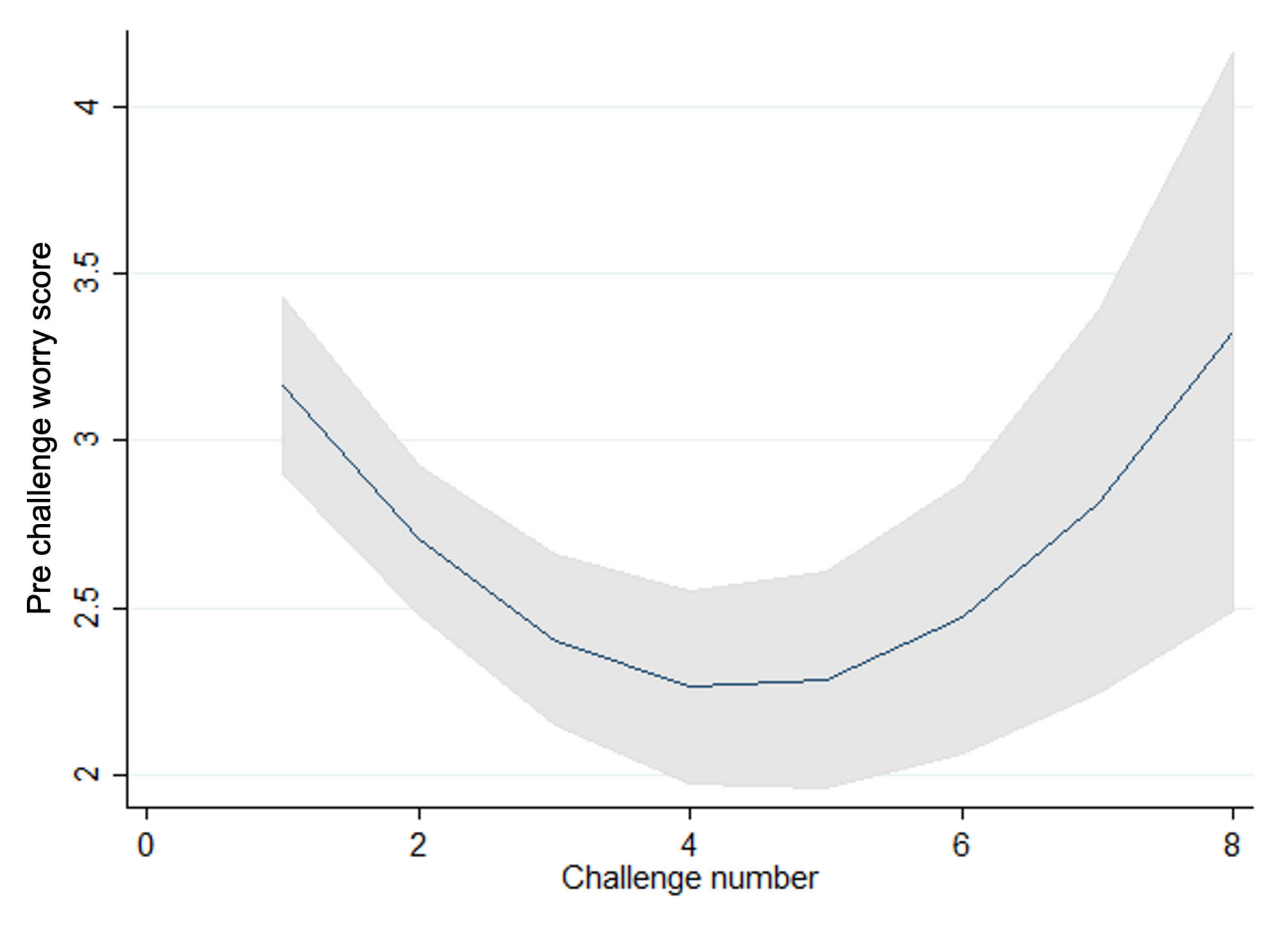
Prechallenge worry scores and challenge number.

### Quantitative Analysis

Recruitment and retention data were collected in line with the CONSORT (Consolidated Standards of Reporting Trials) guidance [22] and are represented in Figure 2. Descriptive statistics were used to describe the participant sample in terms of age, gender, and ethnicity. Data collected was designed to aid understanding of the patterns of usage and engagement and was aligned to the AMUsED (Analyzing and Measuring Usage and Engagement Data) framework for digital interventions [23]. The AMUsED framework checklist (see Checklist 1) was drafted collaboratively by the study team, with statistics developed and completed by the lead statistician (LAC).

Key outcome data focused on anxious expectations using pre- and postexposure worry scales embedded in the game; Goal-based Outcome (GBO) measures captured by the platform; and parent-reported Child Outcome Rating Scale (CORS) to score the parent’s perception of the impact of their child’s anxiety. Usage and engagement data captured both parent and child engagement including: proportion of parents or guardians who shortlisted goals at the start of the study; proportion of parents or guardians who supported children with at least 2 real-life exposures; proportion of parents or guardians able to complete the CORS at start and end of the children and young people’s study engagement period; and the proportion of children completing 3 or more challenges within the game. These data were described using summary statistics. Nonlinear time trends in worry, GBO, and CORS measures were estimated using random effects models. The covariate “Time” corresponded to follow-up timepoint for GBO and CORS. The worry outcomes were collected each time a child/young person completed a challenge, and so “challenge number” was used to reflect time for this outcome.

The following data were collected within Lumi Nova: worry scales pre (eg, “how worried do you feel about the challenge you’re about to take on?”), post (eg, “how worried did you feel during the challenge?”), and future (eg, “how worried would you feel if you had to do it again?”) challenges, which focused on the level of worry experienced by a child; GBO measures, which were child-rated and captured the child’s progression toward achieving their long-term goal; and CORS, which were parent-rated and captured the parent’s perception of the impact of their child’s anxiety.

Data for target outcomes were categorized into:

1. Overall: children who completed more than 3 challenges (could include repetition of the same challenge); reduced anxious expectations measured using pre- and postworry scales scores; improvement in GBOs and CORS.
2. Access: professionals can identify and provide access to children, and children are able to access Lumi Nova on a mobile or tablet device.
3. Use: the proportion of parents or guardians who are able to shortlist goals when accessing the intervention initially, support children with at least 2 out of game exposures, and complete CORS at the relevant time points. Recruitment and retention participant data was collected and analyzed to help assess the feasibility of a future larger study. Qualitative analysis was used to explore the experiences and perspectives of using Lumi Nova. Semistructured interviews were conducted with 11 professionals, 7 children, and 7 parents to explore the usability and acceptability of Lumi Nova and any barriers and enablers to the implementation of the digital technology.

### Qualitative Analysis

Interviews were structured around topic guides developed by the research team based loosely on components suggested by the Nonadoption, Abandonment, and Challenges to the Scale-Up, Spread, and Sustainability (NASSS) framework (see Multimedia Appendix 1 for example guide) [24]. The framework enabled exploration of the potential and challenges of implementing Lumi Nova. The topic guides were flexible to allow new themes to emerge from the interviews. We interviewed children and parents following their engagement with Lumi Nova to explore usability and acceptability, barriers, and enablers to uptake. We interviewed mental health service professionals to understand the implementation barriers and enablers and any unintended consequences they experienced with the implementation of Lumi Nova. Data saturation was reached after interviewing 7 parents, 7 children, and 11 service professionals.

Interviews with staff, children, and mental health professionals were audio and video recorded, transcribed, and analyzed using NatCen Framework Analysis methodology [25,26] in Nvivo 14 (Lumivero) [27]. Quality and credibility of the analysis were ensured by triangulation of the qualitative and quantitative data. One research associate undertook the following process for all interview transcripts.

1. Familiarization of the data
2. Construction of the initial thematic framework through preliminary coding
3. Line-by-line coding and indexing of data under themes and subthemes
4. Creation of framework matrices by combining the matrices from each researcher
5. A second research associate undertook the process for 10% of transcripts to ensure reliability. Interpretation of data was conducted by reading through each theme and summarizing the usability, acceptability, barriers, and enablers to uptake from the perspective of each stakeholder group.

### Ethical Considerations

The study was granted NHS ethical approval (IRAS ID 313721; 22/WM/0121) by West Midlands-Black Country Research Ethics Committee.

## Results

### Quantitative Analysis

Quantitative analysis explored recruitment data and target outcomes of access and use of the Lumi Nova therapeutic game by children and their parents or guardians.

#### Recruitment and Retention Data

In total, 293 children across the participating services were assessed for eligibility; of these, 180 were excluded (reasons shown in Figure 2), and 113 were consented into the study. They included children from low-income households (32 from the lowest 10%, 49 from the lowest 20% levels of deprivation, 14 from the lowest 30% and 18 received free school meals); aged between 7 and 12 years, 32 out of 113 children were aged 10 years and 30 out of 113 children were aged 11 years. There were almost equal numbers of girls and boys who participated, and 89 out of 113 children were White British (see Multimedia Appendix 2 for full demographic characteristics and Multimedia Appendix 3 for the comparison of study sample ethnicities to the population of the local areas).

#### Target Outcomes: Access

In total, 98 of the 113 (87%) accessed the intervention, indicating that professionals could identify and provide access to service users and that service users were able to access Lumi Nova on their mobile device.

#### Target Outcomes: Use

All parents who accessed the intervention were able to shortlist 3 goals for their child from a set of 15 goals, as this is a requirement before children can start using the game. All parents were also required to complete baseline CORS before receiving their game key to access the intervention. The number of challenges completed varied considerably by child, ranging from 0 to 20, with a median of 3 (IQR 1‐5). 19 out of 98 (19%) children did not complete any challenges. 48 out of 98 (49%) children engaged with the predefined minimum exposure dose by completing more than 3 challenges within Lumi Nova. This included repetition of the same challenge, encouraged in graded exposure therapy. Alongside in-game challenges, children were given out-of-game exposure tasks to complete with the support of a parent or guardian. Parents or guardians were required to unlock and validate real-life challenges to ensure safeguarding with a game key. These tasks were engaged with less frequently than the in-game challenges, with 60 out of 98 (61%) children not completing any out-of-game exposure tasks. Although the number completed was again varied with some children completing as many as 10 out of game exposure tasks, 18 out of 98 (18%) children completed only 1 task with the support of their parent or guardian and 20 out of 98 (20%) children had support to complete 2 or more out of game exposures demonstrating the transference of learning to self-manage their worries from digital to real-world practice.

#### Target Outcomes: Outcome Scores

**Figure 4.**
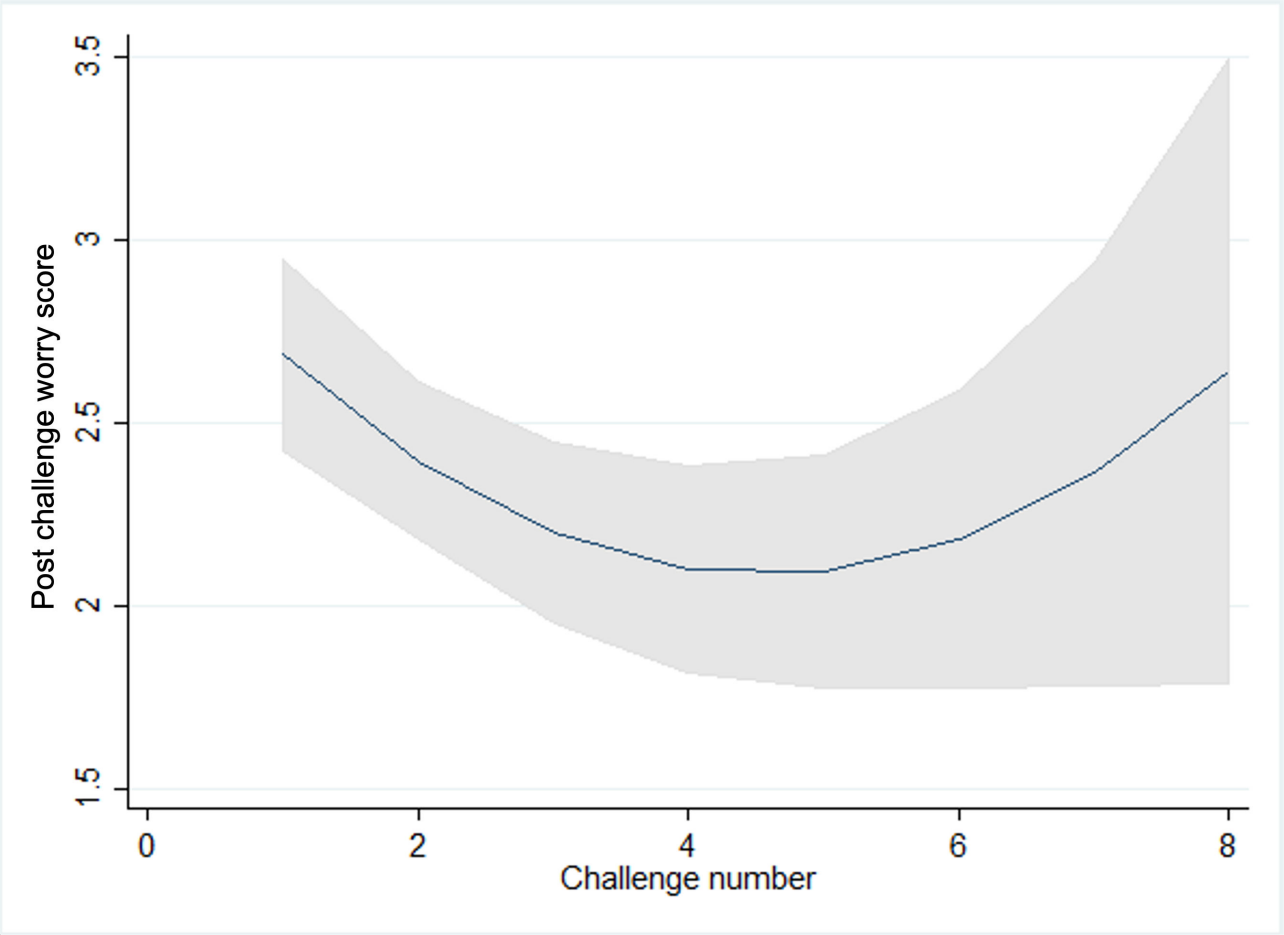
Postchallenge worry scores and challenge number.

**Figure 5.**
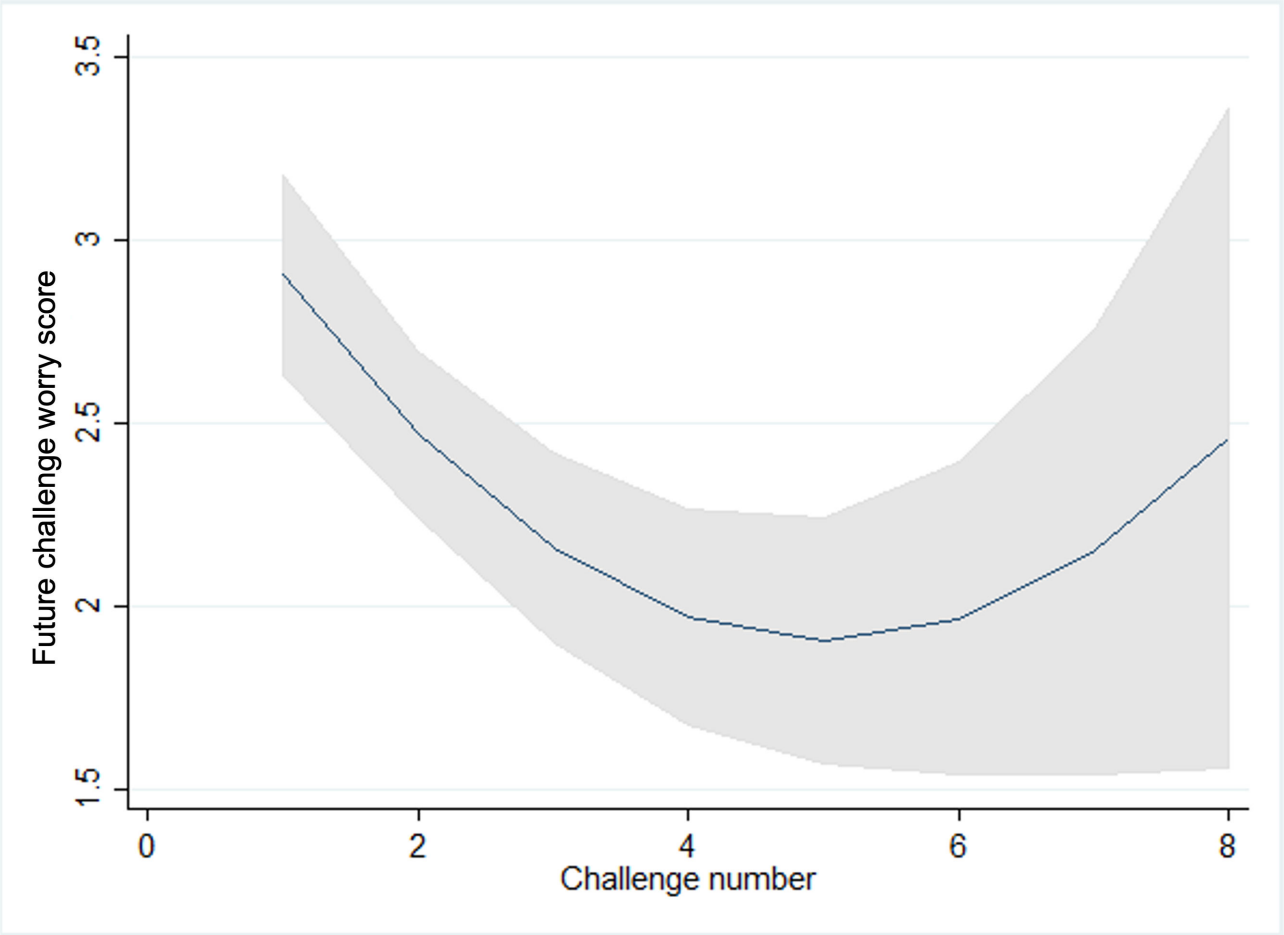
Future challenge worry scores and challenge number.

**Figure 6.**
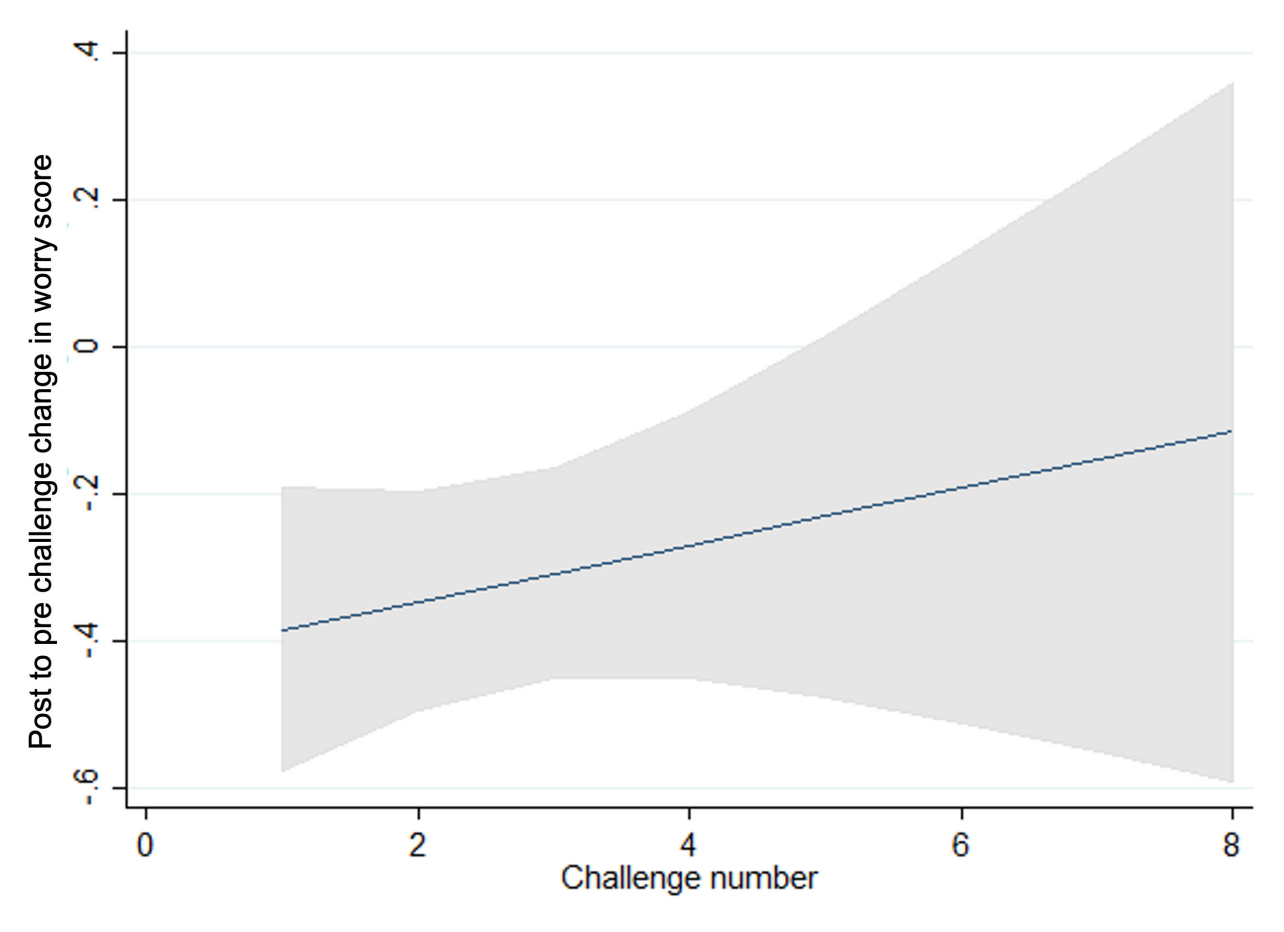
Changes in pre- and postworry score and challenge number.

**Figure 7.**
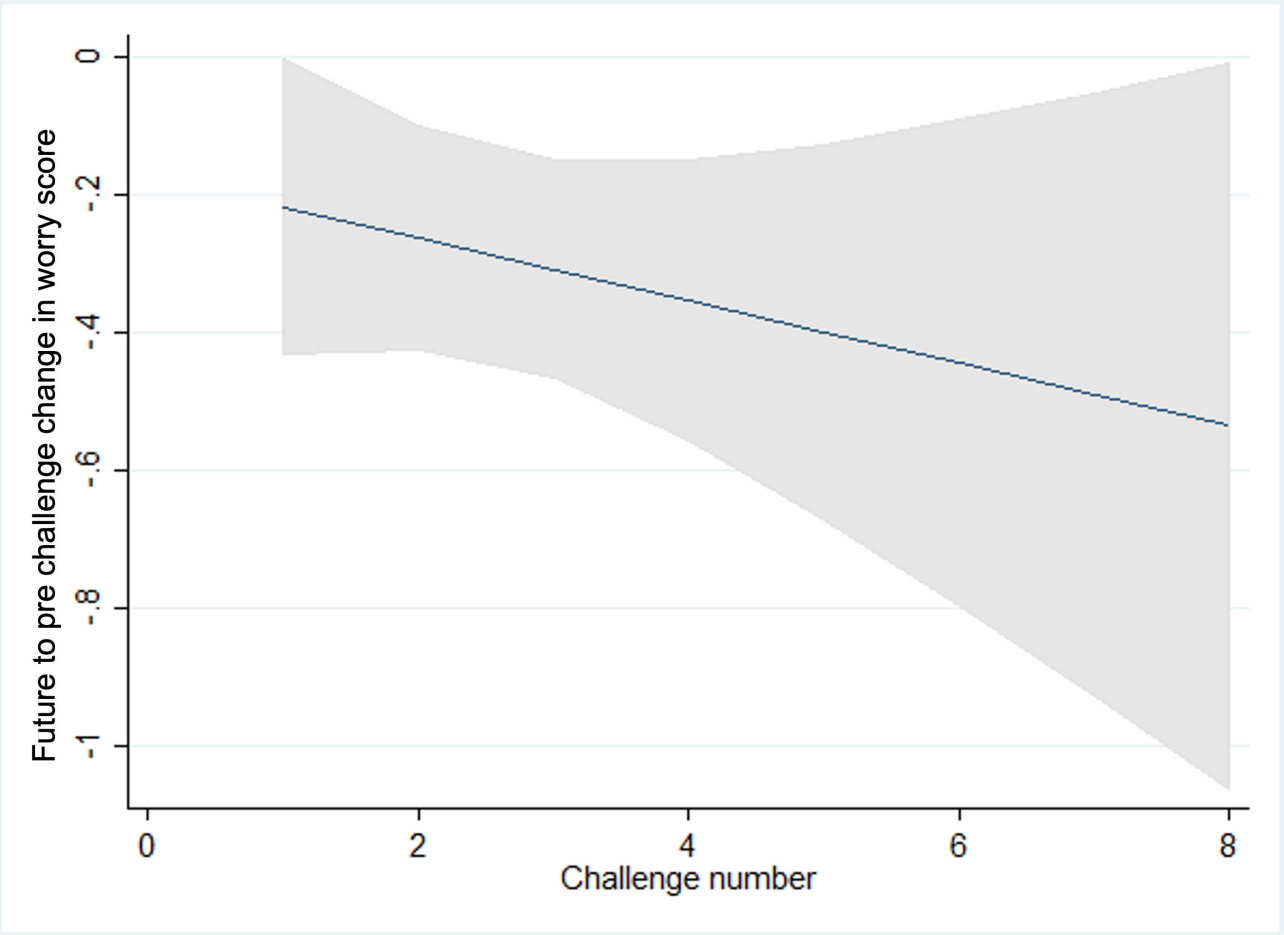
Changes in future and prechallenge worry scores and challenge number.

We observed statistically significant trends in pre-, post-, and future-worry, with scores reducing as children and young people progressed through challenges and increasing again for the latter challenges within a goal in line with what would be expected within graded exposure therapy as the intensity or difficulty of the challenges increases as the child progresses (Figures 3-7). Very few participants completed these latter challenges; however, and so trends are difficult to estimate with any precision. On average, we observe a reduction between pre- and post- and future-challenge worry scores, indicating an improvement in worry following each challenge. This difference is relatively stable across challenges, with no statistically significant trends over time. On average, future worry scores were lower versus prechallenge, and this reduction increased in magnitude as children and young people progressed through challenges, indicating that children and young people felt less anxious undertaking similar challenges in the future.

The number of CORS returned was very variable, with very few parents completing weekly for 6 weeks. Parents completed only two CORS on average, including baseline (IQR 1‐4); however, the date of the last CORS is roughly aligned with the date of the last engagement from children for many people. This makes estimating trends in CORS across the weeks very difficult. On average; however, we saw a 5.2 point improvement in CORS comparing final measurement to baseline (IQR -1.3, 9.8), with 72 out of 98 (73%) parents reporting an improvement in scores.

#### Engagement With in-Game Content

Lumi Nova content can be broken down into “therapeutic” and “non-therapeutic” elements. The therapeutic elements include psychoeducation and exposures, along with the expectation and reflection worry questions. The nontherapeutic content includes the tutorial, dungeon, and wardrobe gameplay. On average, children engaged with therapeutic content for 22.5 (IQR 12.9‐37.1, range 0.1-92.5) minutes, with the most amount of time engaged in exposure elements (median 13, IQR 8‐27.1 minutes). Nontherapeutic gameplay was engaged with for an average of 35.8 (IQR 16.5‐80.7, range 0.2-323) minutes, with an average of 35.3 minutes spent in dungeons.

The duration of use was varied in the sample, and we investigated whether this was associated with any child characteristics. Total session time, time spent in psychoeducation, and time spent in exposures were significantly associated with age, with less time associated with older children. Although the amount of active time spent in dungeon gameplay was not associated with age, older children were less likely to engage with this element at all compared to younger children. There were no statistically significant differences in engagement between genders.

We observed a relationship between engagement and final parent-reported CORS, with more time spent in psychoeducation and exposure elements significantly associated with better outcomes.

A full description of engagement can be found in the AMUsED framework checklist in Checklist 1.

### Qualitative Analysis

Qualitative interview data were analyzed using the NatCen Framework Analysis methodology [25,26] in Nvivo 14 [27]. The thematic analysis was underpinned by the study aims and focused on the usability and acceptability of Lumi Nova to children, their parents or guardians, and mental health professionals. Key themes identified in the data are described below.

#### Usability and Acceptability

Overall, Lumi Nova was reported to be usable and acceptable to economically disadvantaged children and their parents, and they felt it helped children overcome their worries. Most children (5/7) interviewed wanted to continue playing the game after their 8-week access period. Children and young people, parents, and practitioners all reflected that Lumi Nova was helpful in managing anxiety as indicated below.

*[Interviewer]: …So, you think…[Lumi Nova has] helped [reduce feelings of worry]? CYP (child and young person) 7: Yes.*

*Parent 1: … [Lumi Nova] is the only thing that I can associate with the change in CYP1, because before he started the Lumi Nova, he had been doing pretty well with his anxiety…and we started having issues again, [but since playing Lumi Nova] I think he’s no longer seeing his counselor at school, which has been a big deal for him…*

*Practitioner 9: … [one parent] said that [their child] wouldn’t have dealt with [an anxiety inducing] situation in [a well-managed] way. If they hadn’t used Lumi Nova, the experience would be very different [implying the child would not have been able to manage their anxiety]..*

Practitioners felt anxiety would generally become more prevalent in the group of 7‐ to 12-year-olds for reasons such as school transitions, and potential impacts of COVID-19 and social media exacerbating feelings of anxiety, for example, busy places and compulsive behaviors relating to cleanliness.

*Practitioner 8: I think that’s definitely one that I've noticed since COVID, is that fear around busier spaces, crowded spaces, social environments.*

By being able to offer a low-level intervention, practitioners felt children and young people would be empowered to self-manage their anxiety, without the need of a mental health professional.

*Practitioner 2: I think it’s very engaging for children. It’s promoting the idea of self-help, rather than you’re broken and you need an outside specialist to fix you, which I think is a lot of the perception at the minute.*

The majority (6/7) of parents would recommend Lumi Nova to others and suggested that if Lumi Nova was recommended by schools, general practitioners, and other parents, it would be seen as a trusted source of support, as suggested in the quote below:

*Parent 5: I think somewhere like school or other professionals that you come across would make you trust it more, because you think they’ve heard of it from another professional, or it had been recommended. Or another parent that maybe has similar issues is always good, when you hear [by] word of mouth and people say, have you tried this?*

Two out of seven children said they would recommend Lumi Nova to other children. One child explained they would not recommend Lumi Nova to their friends because:

*CYP2: Not many of my friends are the same as me. Not that I know of, anyway.*

Both parents and practitioners reflected on the accessibility of Lumi Nova in terms of simplicity to use and improving access to appointments for families.

*Parent 3: Again, it was simple to follow instructions, download this, click here, job done. Any problems, ring this number.*

*Practitioner 1: …it’s better than attending an actual appointment and less taxing and less time consuming than going into a service and attending an hour-long appointment. And you can fit it into your week easier than you can if you’re relying on a service giving you a set appointment time at that time.*

Parents and practitioners also reflected on progress in terms of the child being able to better manage their anxiety, which was attributed to Lumi Nova.

*Parent 6: I was really quite happy with it, and because, obviously, the progress that CYP6 did make through that, and he does feel a lot more confident in [managing his anxiety].*

*Practitioner 9: …mum said, if they hadn’t [engaged with Lumi Nova], they dealt with the situation completely differently, and she attributed the child’s positive response, and how he dealt with the situation, down to Lumi Nova.*

#### Usage and Engagement

Often, children used their parents’ devices to play Lumi Nova and occasionally had access to their own devices. However, this also proved challenging when parents were separated, as the child was only able to access Lumi Nova when they were with the parent whose device it had been downloaded to.

*Parent 4: Because CYP4 goes to dad’s house half of the time with me and then half with dad, [it’s] because it was on my phone, we then couldn’t send it to dad’s house. So it could’ve been helpful with having two activation codes, couldn’t it?*

One of the recommendations from parents and practitioners was for Lumi Nova to be able to be accessed across multiple devices, perhaps via a login.

*Parent 5: it would have been good to be able to access it on different devices, so he could take one out or, things like that.*

*Practitioner 9: It can only be downloaded to one device, and this device, the screen actually cracked, and… they couldn’t use the device anymore, it had to be sent off for repair. So, any progress had been lost.*

Most parents reflected on needing to support their child with Lumi Nova, and that this was a positive element of the app, as it helped them to better understand their child’s anxiety.

*Parent 6: So that’s what he drew, people bumping into him so then I found out that it was that and I understood that a bit more.*

Practitioners felt that some parents did not have the capacity to support with the game, but explained that when parents did support their child, engagement was generally better and more sustained. From the perspective of practitioners, retention of children to play Lumi Nova was higher when parents were more involved.

*Practitioner 2: …if the parent’s not there, encouraging them, to hang on, let’s take on board this information, let’s think about how you relate this information and reflect on it, they’re going to feel like they’re not getting much out of it.*

*Practitioner 6: …they don’t have that motivation themselves, especially if they’re struggling. And if parents aren’t then helping them out with that then it makes it quite difficult.*

Contrary to this, most children enjoyed playing the game independently with little involvement from parents.

*Pa*rent *3: I’ve not really seen much of it, to be honest. She’s quite independent, so I put the key in, I’ve chosen challenges, and then she’s just snatched it off me and gone off.*

#### Clinically Relevant Outcomes for Economically Disadvantaged Families

Some practitioners suggested parents might:

- Not have enough time to support their child with Lumi Nova;

*Practitioner 11: …in terms of the young people you’re working with, it might have a factor on the availability and time availability of other parents because of the situation they might be in and work commitments or their life commitments and work life balance…*

- Have multiple children or large families;

*Practitioner 5: …sometimes they come from large families with lots of siblings…*

- Live in single-parent households.

*Practitioner 5: …financial situations of families and I know these are systemic difficulties but sometimes these are feeding into young people’s own anxieties, what’s going on financially and family difficulties, separations.*

Factors such as these made it challenging for parents to spend time daily with their child on Lumi Nova.

#### Recruitment and Retention

Younger children seemed to find the game more engaging than older children, which triangulated with the quantitative data.

*Parent 2: …when I asked her what do you mean by that and she went, I mean it’s babyish for me…*

*Practitioner 10: I’d say even from nine, from year four, five upwards to year seven… Although I’ve had one year seven really engage very well with it. The majority were saying I’m bored of it, was what they were saying, after a few [times of playing Lumi Nova].*

Alongside limited support from parents and maintaining engagement, practitioners identified the following barriers to usage:

- Difficulties promoting in schools as staff had limited capacity; however, this was overcome by the team developing a shorter referral form;

*Practitioner 1: …schools are so busy and they have a lot going on. And getting that chance to sit down and digest the information, understand what Lumi Nova is, who it’s aimed for, who they can refer for, for that study and having time to digest that and then get the referrals in to meet that criteria, might be initially quite complex for them.*

*Practitioner 1: …we’ve created a shorter Lumi Nova referral form as well, which I think helps them be able to think, okay, we can quickly bang that in…*

- Difficulties onboarding clinical teams due to their limited capacity and time;

*Practitioner 2: …if they’re going to agree to something like this, they’ve got to agree to giving staff time to get on board with it. And not think, oh well it can be an add on to what they’re already doing.*

- Some families expected a face-to-face intervention;

*Practitioner 4: I think some parents have a bit of resistance to a digital therapy, and…they want a person.*

- Coexisting difficulties that affect a child’s ability to engage with Lumi Nova;

*Practitioner 10: I think there’s a lot of neurodevelopmental concerns. Children who’ve got autism often coexist with anxiety. I think [coexisting difficulties are] really difficult.*

*Practitioner 11: One that we see quite often is low mood and feeling quite low and depression really. So that would obviously impact motivation to engage in the game…*

- Some children have problems that may be too complex for Lumi Nova, that is, above the clinical threshold for the intervention.

*Practitioner 8: …when there’s other complexities, or maybe things in the family, or something like, whether it’s previous trauma, or anything like that, I think that’s where we sometimes find it a little bit difficult to decide, actually, was that appropriate, if it wasn’t just an underlying anxiety, but there’s also lots of other complexities…*

Enablers to usage from the perspective of practitioners were children being supported by parents to play Lumi Nova, and practitioners being supported by the team to overcome any technical difficulties that arose.

*Practitioner 2: …the training was great, so when we did do it, you could see the enthusiasm coming from the Lumi Nova team. It’s people’s mindset when they [are] going into it, I suppose.*

## Discussion

### Principal Findings

Setting up and using the Lumi Nova app in a relatively economically disadvantaged group of families and children was straightforward for most participants. Most children assigned to Lumi Nova succeeded in accessing the intervention and configuring 3 goals. These are the first steps in setting up the game on the mobile or tablet device before use and were completed by nearly all children and families.

This study was designed to allow in-study changes to the digital intervention based on feedback from interviews (Multimedia Appendix 4). These new adaptations were included in the most up-to-date version of Lumi Nova, demonstrating the agility of digital therapeutics in responding to user needs and feedback.

Most children interviewed wanted to continue to use Lumi Nova after the study period had ceased. Practitioners and parents commented on the ease of use of the app after initial setup. Parents and practitioners also generally supported the idea of a mobile game to help with children’s anxiety and were broadly positive about using Lumi Nova. Many practitioners felt that if children were supported by parents whilst playing Lumi Nova, it enabled increased engagement with the intervention. However, they also recognized that this may not be feasible for all families depending on individual circumstances: deprivation is associated with single-parent households [28] and practitioners noted that these families may have limited time available to support children with Lumi Nova.

This study involved a supported delivery model for recruitment of children, with support from mental health teams for onboarding of children in economically disadvantaged areas. The study protocol required practitioners to assess each child before offering the intervention. However, after the children were consented and set up on the game, there was limited involvement from the Education Mental Health and Well-being practitioners and CAMHS staff working in child and adolescent mental health services. We found that Lumi Nova can be used by families without intensive support from mental health professionals in NHS CAMHS. This provides children with the opportunity to self-manage anxiety and worry without necessarily needing to access CAMHS, which would reduce waits and health care resource use, and save families time. Practitioners and parents indicated that children could be supported by relevant educational professionals to use Lumi Nova during school time if parents were unable to support their child at home. This links in with one of the benefits of Lumi Nova being designed for use in naturalistic settings, that is, at home, making it easier for families to access the intervention without needing to travel to clinical settings.

The frequency and duration of usage varied across children, as did the activities completed within the game: almost half (49%) completed 3 in-game challenges. Total session time, that is, time spent in exposures and psychoeducation, varied significantly with age, with less time associated with older children. However, as older children typically have a faster reading speed, this does not necessarily mean that older children were less engaged. Qualitative findings showed that some practitioners and parents noticed improvements in the child’s feelings of anxiety and worry. One parent suggested that additional reminders would have helped them prompt their child to access the intervention and engage with Lumi Nova. There remains some uncertainty around the optimal duration of use; future clinical effectiveness research should aim to identify an optimal “digital dose” of the intervention.

This study was not designed to evaluate the clinical effectiveness of Lumi Nova. However, we did include criteria that would be useful for demonstrating its feasibility in a future randomized control trial (eg, rates of recruitment and retention; implementation barriers and enablers). Here, changes to anxiety levels (clinical outcomes) were not the focus; and, although some anxiety measures were collected and analyzed, limited data meant that we could not interpret these as clinically significant. There was a relationship observed between engagement and final parent-reported CORS, with more time spent in psychoeducation and exposure elements significantly associated with better outcomes. However, while this is promising, it is not necessarily evidence of the intervention’s effectiveness. In addition, as this is a parent-reported outcome, it may be biased where parents who are more engaged and encourage their children to play for longer are more likely to score their child as having better outcomes. Our primary aim was to understand whether it would be suitable for a deprived population of young people, often overlooked in digital mental health studies.

Families who used Lumi Nova with children toward the older end of the 7‐ to 12-year age bracket felt the app was better suited to younger children, that is, 7‐ to 9-year-olds. In some cases, this meant older children were less engaged with the content, which could explain the decrease in engagement over time. However, in one instance, the child explained that the lack of age-appropriate content led to them spending more time focusing on the psychoeducation and exposure aspects of Lumi Nova, as opposed to the gameplay. Arguably, these are the most important elements of the app targeted at improving the child’s ability to manage feelings of worry and anxiety. Seeing Lumi Nova as a fun activity with which to engage rather than a traditional talking therapy is an important element of the design and its success. Subsequent research should adopt a co-design approach to identify requirements and updates that are relevant to older-age children and young people. This may improve their engagement with the intervention.

Literature within this area suggested that studies do not often focus on working with economically disadvantaged children and young people. One of the main reasons for this is the problems of digital exclusion, that is, lack of access to devices, data plans, and low technical literacy. These problems of digital exclusion are often reported to be higher in economically disadvantaged areas [17]. Contrary to conclusions from the literature about accessibility and digital poverty in areas of high deprivation, this study found no barriers for families in accessing Lumi Nova in terms of internet access, mobile data, device access, or technical skills. In several instances, the child had their own device on which to use Lumi Nova. Offering Lumi Nova in other languages, such as Urdu, could have improved accessibility in areas where we delivered the intervention. Language and translation barriers may need greater consideration in similar populations. Therefore, findings from this study suggest the problems of digital exclusion in economically disadvantaged areas may be less prevalent than the literature suggests.

### Limitations

The current study had a number of limitations. The level of deprivation was defined using the Index of Multiple Deprivation [18], which measures deprivation across broad themes including income, crime, education, health, and barriers to housing. As deprivation scores across all themes are combined, high deprivation in one theme can “cancel out” low deprivation in another theme, leading to the possibility of misrepresentation of deprivation. Although recruitment addressed one area of underrepresentation in digital mental health studies (economic disadvantage), the recruited sample lacked ethnically diverse children and families (for ethnicity statistics of the regions and ethnicity of sample, see Multimedia Appendix 3). Future research should aim to include a larger, more diverse, representative sample to improve the broader applicability of findings and address ongoing challenges of underserved populations.

In this study, Lumi Nova was used within a practitioner-led model; therefore, the barriers identified may not apply to families engaging with the intervention independently (ie, self-referral without practitioner support). This model of delivery also required mental health practitioners to assess children, rather than children and families being able to access Lumi Nova freely. Future research could compare both approaches to understand how uptake, usage, and clinical effectiveness of each approach compare.

### Conclusions

Findings from this study challenge widely held beliefs about the ability to deploy digital interventions successfully in economically disadvantaged areas. Children and their families were able to access an appropriate device and data or internet to use Lumi Nova, and they generally reported that they found Lumi Nova usable and acceptable. This study supports widely held calls for more evidence-informed digital tools to support the high demand for children and young people’s mental health services and to provide timely help to children and young people facing common mental health problems. The mode of delivery of these tools requires further exploration. In this study, we focused on a practitioner-supported model. Future studies should explore what mix of practitioner-led, standalone, hybrid model of digital delivery is optimal to deliver support for anxiety problems to children.

Lumi Nova successfully achieved a recommendation by the United Kingdom’s NICE as part of its EVA during the course of the study. The NICE EVA assesses digital products for both clinical effectiveness and value for money. This enables the NHS and patient groups who will benefit from these products to access them sooner. BFB Labs (the developers of Lumi Nova) also improved the software, responding to the feedback received from families and practitioners in this study. Changes to the software included multiuser access on shared or borrowed devices, cloud-saving, and ability to access Lumi Nova via multiple devices (eg, in separated families), greater customization, the addition of voice overs in addition to text, and the ability to save progress more frequently and improved readability (for full details of these changes see Multimedia Appendix 4). It is anticipated that these changes will further enhance the product for disadvantaged children.

## Supplementary material

10.2196/60611 Multimedia Appendix 1 Example interview topic guide.

10.2196/60611 Multimedia Appendix 2 Demographic characteristics of participants.

10.2196/60611 Multimedia Appendix 3 Ethnic groups of study sample compared to area demographics.

10.2196/60611 Multimedia Appendix 4 Lumi Nova features added.

10.2196/60611 Checklist 1 AMUsED checklist.

## Abbreviations

AMUsED: Analyzing and Measuring Usage and Engagement Data
CAMHS: Child and Adolescent Mental Health Services
CBT: Cognitive Behavioral Therapy
CE: Conformité Européenne
CONSORT: Consolidated Standards of Reporting Trials
CORS: Child Outcome Rating Scale
DTAC: Digital Technology Assessment Criteria
EVA: Early Value Assessment
GBO: Goal Based Outcome
MHRA: Medicines and Healthcare products Regulatory Agency
NASSS: Nonadoption, Abandonment, and Challenges to the Scale-Up, Spread, and Sustainability
NHS: National Health Service
NICE: National Institute for Health and Care Excellence
NIHR: National Institute for Health and Care Research
