# Supplementary material for: Implementing a Digital Mental Health Intervention—the Lumi Nova App—to Support Children With Anxiety in Economically Disadvantaged Areas: Mixed Methods Study
Source: J Med Internet Res. 2025 Oct 14;27:e60611. doi: 10.2196/60611 (PMC12520645; doi:10.2196/60611)
Supplement: Multimedia Appendix 1 [file jmir-v27-e60611-s001.docx]

**Example Interview Topic Guide**

**Indicative Topic Guides for Practitioner Interviews**

**(Specific questions may vary slightly from the below)**

Type: Semi-structured individual interviews with 10 service delivery managers

Timeframe: after all participants (for which the individual service delivery manager has oversight) hace completed their participation in the study

Duration: 60 minutes

Led by: GM.Digital Research Associate

Interview Schedule: This guide draws on the NASSS-CAT toolkit: <https://www.phc.ox.ac.uk/research/interdisciplinary-research-in-health-sciences/enasss-cat/enass-cat>

**Intro and Background**

Thanks for your time today. We are here to talk a little bit about your experience of implementing the Lumi Nova intervention. This was the digital intervention supporting 7-12 year olds who have anxiety. Our study, as you will know, was particularly focused on understanding how to implementation this digital mental health intervention in under-served/economically-disadvantaged communities.

Check again that the participant is happy to have the session recorded.

Can you tell us a little bit about your role in the project delivery?

1. **NASSS Area: Illness/Condition**

Questions about anxiety in children in aged 7-12

OVERALL GOAL: to understand if the nature of the illness/condition (anxiety in children aged 7-12) has complexity that has hampered the current implementation study.

- Are there many uncertainties about the presentation of anxiety in children in ways that made this implementation study difficult? (e.g. was it difficult to identify/define?
- Do children with anxiety often/usually have other conditions / co-existing illnesses that might effect their ability to engage with this intervention?
- Do you think this population is likely to change significantly in any way over the next 3-5 years?

1. **The Technology**

Questions about the Lumi Nova technology has affected care

OVERALL GOAL: understand if/how the technology has sufficient complexity to have created barriers to care.

- Were there any problems with the technology that you experienced during the study?
- Did you encounter any technical problems or did Lumi Nova require any significant troubleshooting or support that hampered the implementation?
- Were there any interdependencies/interoperability problems of Lumi Nova with existing IT systems that you routinely use for care of the children enrolled in the study?
- Do you think the technology itself played a key role in the study’s success (or failure)?
- Are there any changed you would recommend to the technology for future implementations?

1. **The Value Proposition**

Questions about the value that Lumi Nova brings from the perspective of this service delivery manager

OVERALL GOAL: understand the value that Lumi Nova can brings or doesn’t bring to different groups of stakeholders

- Do you think Lumi Nova brought value to the children and their parents? Please tell us a little about the value you think it brings/doesn’t bring.
- Do you think Lumi Nova brought value to the clinical teams? Please tell us a little about the value you think it brings/doesn’t bring.
- How do you think Lumi Nova did/didn’t bring value to the Trust? Please tell us a little about the value you think it brings/doesn’t bring.
- Do you have any concerns that Lumi Nova brought negative value (costs outweigh benefits) for some stakeholders?
- Is the value that Lumi Nova brings clear or are there uncertainties around it?

1. **The Intended Adopters**

Questions to understand the target population and the changes it brought them.

OVERALL GOAL: Understand if there is significant complexity relating to the target population of children which is likely to have affected the project’s success/failure.

- Was there complexity in the adoption of Lumi Nova by patients? Can you tell us a little bit about it?
- Was there complexity in the adoption of Lumi Nova by the schools teams? Can you tell us a little bit about it?
- Was there complexity for any other stakeholders in the use of the Lumi Nova to support children with anxiety?

1. **The organisation implementation the technology**

Questions to understand how innovation-ready GMMH were to support Lumi Nova

OVERALL GOAL: Understand if GMMH presented significant complexity that impacted the project’s success/failure

- How ready were GMMH to take on this implementation?
- Would GMMH find it difficult to continue to support Lumi Nova in routine pathways?
- Is the work to embed Lumi Nova into care pathways complete or is there ongoing work that would need to be done?
- Is GMMH likely to restructure in the next 3-5 years in ways that would affect the implementation of Lumi Nova?

1. **The External Context for Innovation**

Questions to understand how the broader context might impact the adoption of Lumi Nova

OVERALL GOAL: to understand the external context and complexity of implementation Lumi Nova

- Can you tell us a little bit about the external context impacted the implementation of Lumi Nova? For example, think about policy, patient organisations, regulatory contexts, commercial contexts and any other external contexts you think are relevant.

**Thank you for your time and participation in this study.**
