# Supplementary material for: Implementing a Digital Mental Health Intervention—the Lumi Nova App—to Support Children With Anxiety in Economically Disadvantaged Areas: Mixed Methods Study
Source: J Med Internet Res. 2025 Oct 14;27:e60611. doi: 10.2196/60611 (PMC12520645; doi:10.2196/60611)
Supplement: Multimedia Appendix 2 [file jmir-v27-e60611-s002.docx]

**Demographic Characteristics of Participants**

| **Age** | **No. of children** |
| --- | --- |
| **7** | 7 |
| **8** | 14 |
| **9** | 24 |
| **10** | 32 |
| **11** | 30 |
| **12** | 6 |

| **Gender** | **No. of children** |
| --- | --- |
| **Male** | 54 |
| **Female** | 59 |

| **Ethnicity** | **No. of children** |
| --- | --- |
| **Asian/Asian British - Pakistani** | 1 |
| **Chinese** | 1 |
| **Mixed, Mixed-White and Asian** | 2 |
| **Other ethnic groups** | 1 |
| **Polish** | 1 |
| **White British** | 89 |
| **Unknown** | 18 |

| **Level of deprivation** | **No. of children** |
| --- | --- |
| **Lowest 10%** | 32 |
| **Lowest 20%** | 49 |
| **Lowest 30%** | 14 |
| **Free school meals** | 18 |
