# Supplementary material for: Implementing a Digital Mental Health Intervention—the Lumi Nova App—to Support Children With Anxiety in Economically Disadvantaged Areas: Mixed Methods Study
Source: J Med Internet Res. 2025 Oct 14;27:e60611. doi: 10.2196/60611 (PMC12520645; doi:10.2196/60611)
Supplement: Multimedia Appendix 3 [file jmir-v27-e60611-s003.docx]

**Ethnic groups of study sample compared to area demographics**

| Ethnic Group | Study Sample | Area 1 | Area 2 |
| --- | --- | --- | --- |
| Asian, Asian British or Asian Welsh | 0.01% | 20.10% | 1.80% |
| Black, Black British, Black Welsh, Caribbean or African | 0.00% | 3.80% | 1.20% |
| Mixed or Multiple ethnic groups | 0.02% | 2.20% | 1.30% |
| White | 78.76% | 71.90% | 95.00% |
| Other ethnic group | 0.03% | 1.90% | 0.70% |

Note ethnicities of 21.18% of the study sample were unknown.
