# Supplementary material for: Implementing a Digital Mental Health Intervention—the Lumi Nova App—to Support Children With Anxiety in Economically Disadvantaged Areas: Mixed Methods Study
Source: J Med Internet Res. 2025 Oct 14;27:e60611. doi: 10.2196/60611 (PMC12520645; doi:10.2196/60611)
Supplement: Multimedia Appendix 4 [file jmir-v27-e60611-s004.docx]

**Lumi Nova Features Added**

Specific changes made as a result of the Agile Software Sprint:

- The wardrobe now shows all the hidden customisation options that are available for players to collect.
- Voice overs are now included and are turned on by default for all players. Voice overs can be turned off via the Settings menu.
- Ability to replay the voice over.
- Cloud Saving has been implemented to allow users to save their progress and play across up to 3 devices using their Game Key.
- Costume rewards are now provided for completing new challenges as well as for completing previous challenges.
- Ability to set up custom reminders to play (in progress).
- Ability to adjust text speed via the Settings menu (in progress).
- Enabling multiple users to gain unique access to Lumi Nova on a single device.

| **#** | **Release No.** | **Feature Added** | **Requirement** | **Stakeholder** | **Main theme** | **Supporting quote** |
| --- | --- | --- | --- | --- | --- | --- |
| 1 | 1.0.4 | The wardrobe now shows all the hidden customisation options that are available for players to collect. | More items/clothes in the store | CYP | Game Improvements | CYP 1: I feel like the store needs a bit more items that you can get if you play the challenges.  CYP7: Different dresses, and stuff. |
| 2 | 1.0.4 | Ability to replay the voice over. | Replay instructions | Parents | Game Improvements | P2: …that first bit there’s a good sentence worth, isn’t there, of information that comes down quite fast, if you replay it… |
| 3 | 1.0.4 | Voice overs are now included and are turned on by default for all players. Voice overs can be turned off via the Settings menu. | Characters to read out instructions | Parents and Practitioners | Game Improvements | P1: I do think maybe a text to speech where it says it to them, so that you have that opportunity to put that on for kids that don't read well.  PRAC3: I think if it was read out, it might be easier, as well as having it there, if it was read out as well. Yes. |
| 4 | 1.0.4 | Cloud Saving has been implemented to allow users to save their progress and play across up to 3 devices using their Game Key. (Requires internet connection) | Being able to play across multiple devices | CYP, Parents and Practitioners | Game Improvements | P4: Because CYP4 goes to dad's house half of the time with me and then half with dad, is because it was on my phone, we then couldn't send it to dad's house. So it could've been helpful with having two activation codes, couldn't it?  PRAC2: I think they’d been doing on mum’s phone, and I think mum’s phone then broke, and they were then trying to download it on a different device. |
| 5 | 1.0.4 | Cloud Saving has been implemented to allow users to save their progress and play across up to 3 devices using their Game Key. (Requires internet connection) | Being able to save the game | Parents | Game Improvements | P4: But then there was other times, wasn’t there, when we’d get to school and he’d be halfway through something and I’m, like, you can’t finish it because you need to go to school, but there was no way of saving it, was there? |
| 6 | 1.0.4 | Costume rewards are now provided for completing new challenges as well as for completing previous challenges. | More/different rewards for challenges - same rewards for completing old goal and new goal | CYP | Game Improvements | CYP6: For doing a challenge you’ve already done you get more rewards than doing a new challenge. |
| 7 | 1.0.5 (due to release mar 2024) | (In progress) Ability to set up custom reminders to play. | Reminders to play at set time | Parents | Game Improvements | P4: …so if it came up on your phone and said, don’t forget I’m here, you know, it’s your time to play and it went off and nine o’clock every day. |
| 8 | 1.0.5 (due to release march 2024) | (In progress) Ability to adjust text speed via the Settings menu. | Able to slow down/speed up game, and speed of information presented | Practitioners | Game Improvements | PRAC3: The speed, ooh, yeah, yeah, like a little dial. |
