# Supplementary material for: Implementing a Digital Mental Health Intervention—the Lumi Nova App—to Support Children With Anxiety in Economically Disadvantaged Areas: Mixed Methods Study
Source: J Med Internet Res. 2025 Oct 14;27:e60611. doi: 10.2196/60611 (PMC12520645; doi:10.2196/60611)
Supplement: Checklist 1 [file jmir-v27-e60611-s005.docx]

**AMUsED Framework Checklist**

|  | 1. Descriptions of usage variables. Which usage variables are relevant to the intervention and in which format (e.g. number of users/sessions, duration, percentage of total, dichotomous)? | | |
| --- | --- | --- | --- |
| **1.1** | **Completing intervention/trial period (stage1; 1.1 & 2.2).**  E.g. How many users complete the trial?  What is the average time taken to complete? | How many goals are completed    Time taken to ‘complete’ for those completed – days between activation and goal completion    How many complete more than 3 challenges (Target outcomes – use)  Proportion of parents/guardians who are able to support CYP with at least 2 out of game exposures (Target outcomes – use) | Summaries given out of 98 who accessed the intervention   - 6 CYP completed one goal or more (5 (5%) completed one goal and 1 (1%) completed 2 goals). We recognise focusing on goals obscures the progress made by achieving steps towards the overall long term goal, this was a limitation of the data used in this study. - Of those completing one goal, it took on average 39.5 days (IQR 28 – 65, min 17, max 71) - The one individual who completed two goals did so in 47 days. - 49% of CYP have completed more than 3 challenges. - 20% of CYP have had parent/guardian support to complete at least 2 out of game exposures |
| **1.2** | **Logins or sessions where the intervention was accessed (stage 1; 1.1 & 2.2).**  E.g. How many users start/complete each login/session?  How long does it take to complete each session?  How many pages are viewed within the session?  Which session has the highest proportion of pages viewed, or duration of time spent on it? | How many start overall:  How many accessed the intervention?  How many/proportion set up at least 1 goal?  How many/proportion set up 3 goals?    How many ‘sessions‘ – times opened app, not necessarily played/accessed therapeutic content  Total number of unique sessions | - 98 of the 113 (87%) accessed the intervention - All parents were able to set up 3 goals (one set a second set of 3 goals) - Total number of unique sessions: median 13.5, IQR 5 – 24, min 1, max 62. - Data for individual sessions was not available. |
| **1.3** | **Date of login and usage.**  E.g. When do users login? What time of year? Are there changes in frequency of logins? | Data was only available for activation date. |  |
| **1.4** | **Time of day of login and usage.**  E.g. What time of day is usage? Are users more likely to spend longer on the intervention at certain times? | Data for individual sessions was not available. |  |
| **1.5** | **Days/weeks of usage (stage1; 1.1 & 2.2).**  E.g. For how many days/weeks out of the total is the intervention accessed for? How many times within a week is the intervention accessed?  Are there repeated uses within the same day? | Number of unique days played  Number of sessions  Total session time  Time from activation to last play  Data for individual sessions was not available. | - The level of engagement was highly skewed. The average number of days CYP played Lumi Nova on was 5. Though some played on as many as 35 separate days, 30% of users only played on one or two days - Total number of sessions: Even with relatively few days of engagement, users appear to have logged in to play multiple times a day. In those only playing on two days or fewer, there was an average of 3 sessions played      \|  \| Median \| IQR \| Min \| Max \| \| --- \| --- \| --- \| --- \| --- \| \| Number of days played on \| 5 \| 2 – 8 \| 1 \| 35 \| \| Number of sessions \| 13.5 \| 5 – 24 \| 1 \| 62 \| \| Total session time (hours) \| 1.3 \| 0.7-2.3 \| 0 \| 14.4 \| \| Time from activation to last play (weeks) \| 3 \| 0.9 – 6.4 \| 0 \| 17.7 \| |
| **1.6** | **Response to prompts/notifications (e.g. requests to login, email, text, upload data) (stage1; 1.1 & 2.2).**  E.g. How many responses are sent? How long after receiving notification do users take to log-in or respond? |  |  |
| **1.7** | **Features/linked menu components used (stage 1; 1.1, 1.2 & 2.2).**  E.g. How many features/components are accessed?  How many users access each one?  Which are completed and by how many users? Which feature/component has the highest proportion of pages viewed or time spent? What order are they viewed in?  Is this the anticipated order?  Which have the highest proportion of drop-out? | Total time (minutes) interacted with:  Tutorial  Psychoeducation  Exposure  Dungeon  Wardrobe  Anxious expectations screens  Reflections (anxiety post/future measures)  Idle time  Goaway – (Total time spent in the game once 30min limit has been reached and PIP tells player to come back tomorrow in seconds)    Categorised by ‘therapeutic components’ and ‘other/game components’ | \|  \| n (N=98) \| Median \| IQR \| Min \| Max \| \| --- \| --- \| --- \| --- \| --- \| --- \| \| ‘Therapeutic’ \| \| \| \| \| \| \| Psychoeducation \| 90 \| 3.6 \| 2.4-4.7 \| 0.3 \| 16.0 \| \| Exposure \| 84 \| 13.0 \| 8.0-27.1 \| 0.1 \| 52.6 \| \| Expectation \| 86 \| 4.3 \| 2.3-8.2 \| 0.2 \| 23.2 \| \| Reflection \| 80 \| 4.3 \| 2.6-6.6 \| 0.6 \| 26.5 \| \| ‘Non-therapeutic’ \| \| \| \| \| \| \| Tutorial \| 92 \| 11.8 \| 9.8-13.3 \| 0.2 \| 32.3 \| \| Dungeon \| 76 \| 35.3 \| 15.6-83.2 \| 1.2 \| 267 \| \| Wardrobe \| 56 \| 1 \| 0.5-2.9 \| 0 \| 12.5 \| \| Idle time \| 63 \| 2.8 \| 1.1-6.4 \| 0.1 \| 43.4 \| \| Additional gameplay (“Go away”) \| 40 \| 1.1 \| 0.4-3.1 \| 0.1 \| 30.3 \|   All times are reported in minutes    Order of play from setup means 86% of users who accessed the intervention made it to at least the first exposure. Average total exposure time was 13 minutes.    Note: of the non-therapeutic elements, around a quarter of users did not engage with the dungeon/play element, and only 57% interacted with their character in the wardrobe function. This was the least interacted with element, with an average of 1 minute total time spent in wardrobe |
| **1.8** | **Revisiting components/features (stage 1; 1.1, 1.2 & 2.2).**  E.g. Are any used repeatedly?  How many times are they revisited, and for how long?  Which are most revisited? | N/A |  |
| **1.9** | **Type of content/BCTs used (excluding administration pages)** (stage 1; 1.2 &  2.2).  E.g. How many groups of pages with similar content are accessed and by how many users? How many pages within the group are used? How many users view each page?  Which groups of pages have the highest proportion of views?  Which pages are viewed at each login, and when is the largest amount of pages viewed? Which pages have higher drop-out? | N/A |  |
| **1.10** | **Completing ongoing measures** (e.g. monthly questionnaires, response options  within content pages, uploading information or text responses) (stage 1; 2.1 & 2.2).  E.g. How many users complete ongoing measures?  When do they complete them?  Do they also access the intervention at that time? | CORs weekly parent survey  GBO in game Worry (reflections)  Pre-challenge  Post-challenge  Future (i.e. How worried would you feel if you had to do it again) | CORS   - 36% of parents completed only the baseline measurement for CORS (distribution of CORS returned in total), giving no additional follow up measurement. - Of those completing at least one more than baseline, the average number of CORS returned is 3 (IQR 2-5, min 2, max 16).  When we restrict this to be during the 6 weeks of ‘game play window’ as described in protocol (where we would expect 7 CORS returned for ‘complete’ data), we have an average of 3 (IQR 2-4, min 2, max 6) for the 60 individuals returning more than just baseline   GBO   - 11 CYP (11%) did not complete any GBO measures - 17% completed only 1 (baseline) - Of those completing at least one more than baseline, the average number of GBOs returned was 3 (IQR 2-4, min 2 max 6) within six weeks   WORRY   - 79 CYP (81%) completed the first pre/post/future worry measure (goal 1, challenge 1, attempt 1) - Every pre/post/worry measure was completed at each challenge |
| **1.11** | **External device usage (e.g. wearables and other sensor technologies)** (stage 1;  2.3).  E.g. How much time is spent with the device? How many times is It used?  What number of days/weeks is it used for? | N/A |  |
|  | **2. Relationships between usage and participant characteristics. Are users’ demographic, physical or psychosocial characteristics at baseline related to intervention usage?** | |  |
| **2.1** | **Are any characteristics at baseline related to usage?**  E.g. Is anxiety associated with revisiting features? Is current health related to usage of external devices? Are users who spend more time on the intervention older than those who spend less time? Which characteristics are associated with dropout? | Age  Gender  Ethnicity (only 2 non-white CYP)  Disability (only 4 with disability)  Associated with total time spent in  Psychoeducation  Exposure  Dungeon  Wardrobe  Game overall  (Total session time)  Association with engagement (yes/no) with  Dungeon  Wardrobe | \|  \| Age \| \| \| Gender \| \| \| \| --- \| --- \| --- \| --- \| --- \| --- \| --- \| \| Time (mins) \| Coeff. \| 95% CI \| P value \| Coeff. \| 95% CI \| P value \| \| Total session time \| -25.4 \| -42.5, -8.3 \| 0.004 \| 21.1 \| -25.2, 67.5 \| 0.368 \| \| Psychoeducation \| -0.6 \| -1.0, -0.2 \| 0.008 \| 0.3 \| -0.9, 1.5 \| 0.622 \| \| Exposure \| -2.3 \| -4.4, -0.3 \| 0.023 \| 1.9 \| -3.7, 7.4 \| 0.500 \| \| Dungeon \| -5.8 \| -15.7, 4.1 \| 0.249 \| 7.8 \| -18.6, 34.2 \| 0.558 \| \| Wardrobe \| -0.5 \| -1.0, 0.1 \| 0.113 \| 0.7 \| -0.8, 2.2 \| 0.344 \| \| Engagement  (Yes/No) \| OR \| 95% CI \| P value \| OR \| 95% CI \| P value \| \| Dungeon \| 0.6 \| 0.4, 0.9 \| 0.012 \| 0.9 \| 0.4, 2.4 \| 0.893 \| \| Wardrobe \| 0.8 \| 0.6, 1.1 \| 0.099 \| 1.7 \| 0.8, 4.0 \| 0.194 \|   Game play decreases with age  Less likely to play dungeon at all the older a player is (22/98 did not play in dungeon at all). |
| **2.2** | **Do high/low users differ by other usage factors?**  E.g. Do users who spend more time on the intervention view more types of content than users who spend less time? Is usage of an external device related to intervention usage? | N/A |  |
|  | **3. Relationships between usage, behavioural determinants, and target behaviours. Which usage variables are associated with follow-up measures for target behaviour and behavioural determinants? Which usage variables help explain changes in behaviour across the intervention?** | |  |
| **3.1** | **Are baseline measures for behavioral determinants/target behavior related to**  **usage?**  E.g. Is the number of days the intervention is used for related to a behavioural determinant? Do users with low target behaviour spend less time on the intervention | Is baseline (T1) CORS related to  Number of unique days played  Total session time | \|  \| IRR \| 95% CI \| P value \| \| --- \| --- \| --- \| --- \| \| Number of days \| 1.0 \| 0.99, 1.04 \| 0.100 \| \|  \| Coeff. \| 95% CI \| P value \| \| Total session time (mins) \| 1.8 \| -1.3, 4.9 \| 0.243 \|   Incidence rate ratio (IRR) reported for Negative binomial regression  No associations between baseline CORS and time played |
| **3.2** | **Which usage variables are related to behavioural determinants/target**  **behaviours and at follow-up?**  E.g. Do users who view a group of pages  containing a specific BCT score higher/lower for the associated behavioural determinant? Is completing/not completing a particular component associated with target behaviour at follow-up? Is the time spent on a session  related to target behaviour? | Time in ‘therapeutic contents’  Number of challenges completed (total across all goals)  Associated with final CORS  Note, this is not necessarily “Follow up” | \|  \| Coeff. \| 95% CI \| P value \| \| --- \| --- \| --- \| --- \| \| Psychoeducation (mins) \| 0.9 \| 0.3, 1.5 \| 0.004 \| \| Exposure (mins) \| 0.2 \| 0.1, 0.3 \| 0.009 \| \| Number of challenges completed \| 0.7 \| 0.3, 1.1 \| 0.001 \|   More engagement is associated with better outcomes (CORS)  This is not necessarily a sign that the intervention is “effective”. Could be a parent bias – CYP encouraged to use it more by parent and then parent reported outcome. |
| **3.3** | **Is usage associated with measures for acceptability/satisfaction at follow-up?**  E.g. Are high levels of satisfaction associated with accessing more pages?  Do users with low satisfaction spend less time using external devices? | What are satisfaction measures?  Ease of use response from child, where 1 is very easy and 5 is very hard (time-point unknown) | \| Child Ease \| n \| % \| \| --- \| --- \| --- \| \| 1 \| 11 \| 29 \| \| 2 \| 6 \| 16 \| \| 3 \| 12 \| 32 \| \| 4 \| 5 \| 13 \| \| 5 \| 4 \| 11 \|   Only 9 CYP found ease of use hard/very hard, but missing data on 61% of CYP |
| **3.4** | **Do users who report positive changes in behavioral determinants/target behavior from baseline to follow-up use the intervention differently to those who do not?**  E.g. Do users who report positive increases in a behavioral determinant view more pages from a specific component containing an associated BCT? Do users who report positive behavior change spend more time on the intervention? | Behaviour change:  CORS  Final CORS – T1 CORS  Anxiety  Final pre-challenge - Pre-challenge 1  Final post-challenge - Post-challenge 1  Final Future challenge – Future challenge 1  Time on the intervention  Total time played  Time in ‘therapeutic contents’  Number of challenges completed (total across all goals) | CORS  63/98 had at least two CORS measures in which to calculate change  Last CORS rarely in week 6: Time between first and last scores on average 28 days (IQR 13 – 56, min 7, max 137)  This differed in those who report an increase (improvement) in CORS (31 days, IQR 17-59, min 7, max 137, n=31) and those who reported a decrease in CORS (16 days, IQR 8 – 34, min 7, max 116, n=17)  No association between usage and an increase in CORS from baseline (where this change is scored as binary reflecting increase or no increase )    Worry  More challenges completed in those who saw a decrease in future worry (but not pre challenge worry or during challenge reflection on worry) |
| **3.5** | **Are relationships between usage and target behaviour moderated by demographic, psychosocial or health factors?**  E.g. Is the relationship between time spent on the intervention and target behaviour altered when moderated by anxiety? | N/A |  |
| **3.6** | **What level of usage is necessary for ‘effective engagement’?**  E.g. Do outcome measures plateau after viewing certain content, or after a certain amount of time or sessions completed | N/A |  |
